# Clinicians perceptions of a telemedicine system: a mixed method study of Makassar City, Indonesia

**DOI:** 10.1186/s12911-020-01234-7

**Published:** 2020-09-17

**Authors:** Dea Indria, Mohannad Alajlani, Hamish S F Fraser

**Affiliations:** 1 Yorkshire Center for Health Informatics, University of Leeds, Leeds, UK; 2 PO BOX INDRIADEA, Jakarta, JKB 11000 Indonesia; 3 Institute of Digital Healthcare, University of Warwick, Coventry, UK; 4 Brown Center for Biomedical Informatics, Brown University, Providence, RI USA

**Keywords:** Perception, Primary health care, Telemedicine

## Abstract

**Background:**

This case study in Makassar City, Indonesia aims to investigate the clinicians’ perceptions, including both satisfaction and barriers in using telemedicine in a large, established program which supported 3974 consultations in 2017.

**Methods:**

A mixed methodology was used in this research utilizing a questionnaire with 12 questions, and semi-structured interviews. A purposeful sample of clinicians using the telemedicine system at the 39 primary care clinics in Makassar City were surveyed.

A total of 100 clinicians participated in this study. All of them completed the questionnaires (76.9% response rate) and 15 of them were interviewed.

**Results:**

The result showed that 78% of the clinicians were satisfied with the telemedicine system. In free text responses 69% said that telemedicine allowed quicker diagnosis and treatment, 47% said poor internet connectivity was a significant obstacle in using the system, and 40% suggested improvement to the infrastructure including internet connection and electricity.

**Conclusion:**

Overall, the clinicians were satisfied with the system, with the main benefit of rendering the diagnosis faster and easier for patients. However, poor internet connectivity was indicated as the main barrier. Most of the clinicians suggested improving the infrastructure especially the internet network.

## Background

Indonesia is an archipelago country which consists of more than 17,000 islands with more than 200 million people. It faces problem caused by an uneven distribution of health professionals, and healthcare disparities due to a vast territory and transport challenges [1, 2]. A study from the World Health Organization (WHO) showed that 18,377 doctors worked in the Java and Bali islands, whereas 15,359 doctors worked in the rest of the country, an overall rate of only 13/100,000 population [2]. To access certain specialist physician, patients from remote areas need to travel long distances which can cause delays and high costs in accessing the health service.

To overcome this problem, Indonesia began to develop telemedicine, since it enables health professionals to communicate over long distances using the internet for the exchange of health information [3, 4]. It is believed that telemedicine has the potential to overcome healthcare disparities and improve equitable access to healthcare by receipt of specialist’s second opinions from a distance, and could also reduce workload and professional isolation of remote healthcare workers [4, 5].

The growth of telemedicine in Indonesia began in 1985, but unfortunately most of the telemedicine activities in Indonesia are not well evaluated and documented due to the limited number of studies [1]. One of the most successful telemedicine systems in Indonesia is the system used in Makassar City, the capital of South Sulawesi Province. However, evidence regarding its functioning is still limited, especially regarding the clinicians’ perception.

Clinician’s perception was chosen as the research question because of the critical role they play in the health system and the functioning of telemedicine. Their acceptance is important to increase positive attitude and confidence in using telemedicine, for local sustainability and the further deployment of the technology [6].

The driving force behind this research was the lack of studies conducted in this country on telemedicine, and the need to identify barriers and problems that could prevent wider and more effective telemedicine utilization in Indonesia.

## Objectives

To understand how well telemedicine actually works from the providers’ viewpoint and how its effective use and impact could be increased in Indonesia. Therefore, this research aims to investigate their satisfaction, perceived advantages and barriers, and suggestions for future improvement.

## Telemedicine in Makassar City

In 2014, Makassar successfully developed a Makassar Telemedicine System to provide a free telemedicine service. The telemedicine system is operated by 44 primary care centers in the mainland serving 1,710,114 people, and 2 in the surrounding islands in the Makassar Strait serving around 10,000 people [7].

**Fig. 1:**
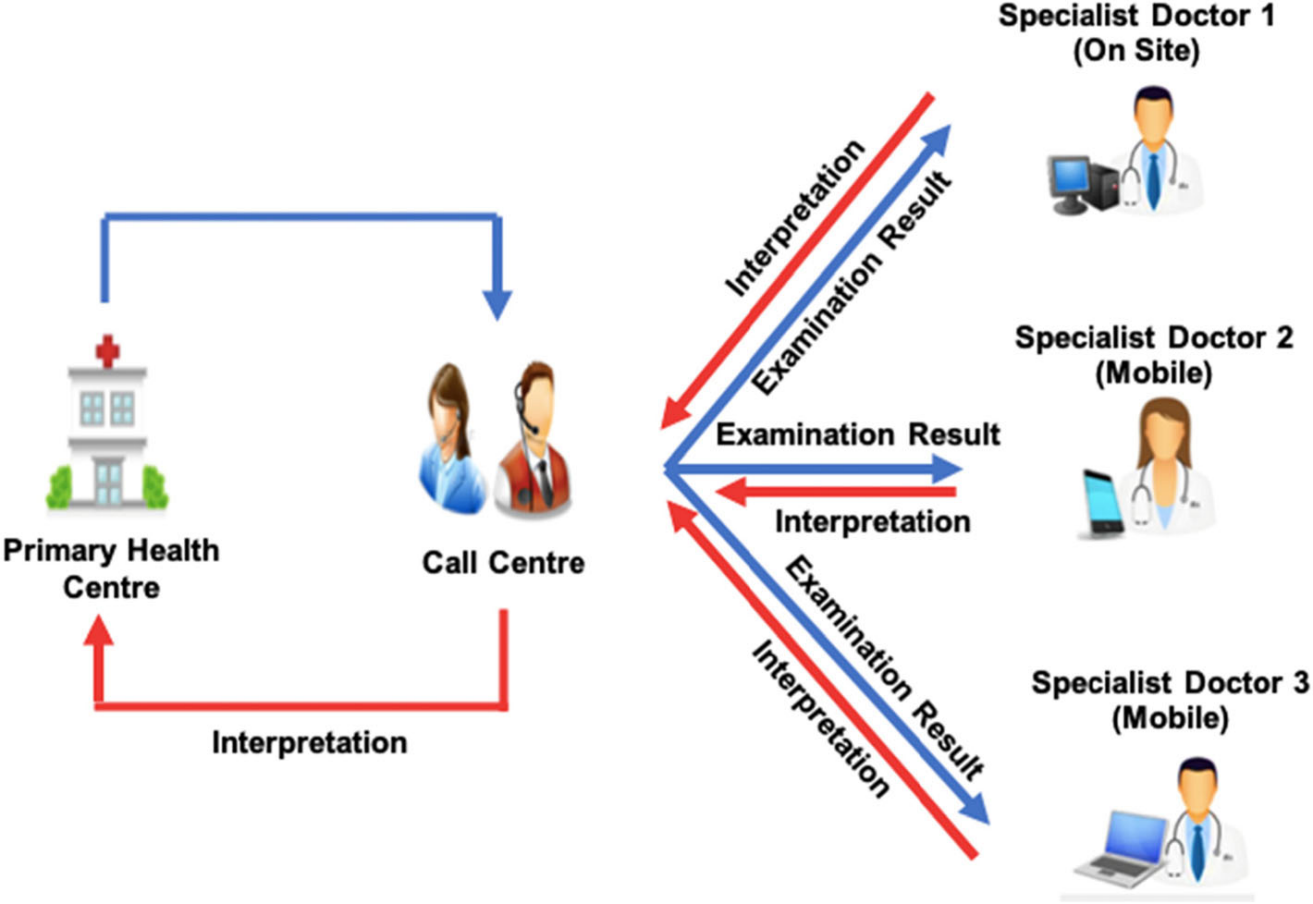
Telemedicine System in Makassar City [7]

The system used is store-and-forward telemedicine to seek a second opinion from a specialist for two types of services: tele-electrocardiography (tele-ECG); and tele-ultrasonography (tele-USG). Tele-ECG is used for early diagnosis for patients suspected to have heart diseases, and for follow-up examination. Tele-USG is currently used for obstetrics only. The tele-ECG or tele-USG machine records patients’ data and the clinician in the primary care clinic uses the application to send the information through the internet to a specialist. The specialist receives notifications, and can then access the patients’ data anywhere using their smartphone or other gadgets as long as they have internet access. The local Government established a cooperation with the local University to develop the system, and as the control center to monitor the traffic of sending and receiving communication in the system (See Fig. 1) [7]. In 2017, there were 3974 consultations, with over 8000 consultations since the project start (see Additional file 1).

## Methods

### Study design

This research followed a mixed methodology approach with questionnaires and interviews in Indonesian as data collection methods. A purposive sampling technique was used. The “clinicians” in the context of this study were general practitioners, nurses, and midwives that were using telemedicine equipment in primary care centers in and around Makassar City. Thirty nine out of 46 primary care centers agreed to participate in the study. All sites started using the system in 2014 and the questionnaire was administered in mid of 2017.

Most sites had 4 or more cases a month, and there were sites with more than 10 cases a month (see Additional file 2). Staff therefore had regular experience of using the system. Staff from all 39 primary care centers returned the questionnaires, all these sites regularly carried out teleconsultations.

### Participants

To recruit participants for this study, invitation letters were sent to 46 primary health centres. However, only 39 primary health centres participated in this study.

### Survey

The questionnaire consisted of 12 questions to assess clinician’s overall perception of using telemedicine. The last part consisted of three open-ended questions related to aspects of the service that they liked or disliked as well as suggestions for future improvements. A year before this study, the lead author visited Makassar city to observe their telemedicine system and interview a group of doctors. Questions for questionnaire and interview were chosen based on those observations and interviews, and extensive literature review [8–10]. They were translated into the Indonesian language.

### Interviews

The semi-structured interviews were conducted one to one, face to face in a practice-room with a relaxed atmosphere without the presence of other people and started with an explanation regarding the reason of this study. During the interview, audio recording was used so the interviewer could listen attentively without distraction. This one-time interview consisted of seven questions to discover issue relating to their experience in using telemedicine, examine the possible benefits and barriers they faced, and their opinions on future improvements. The transcript was not returned to the participants to seek feedback.

In-depth interviews were conducted with 10 general practitioners and 5 nurses. The interview subjects were chosen because of their seniority and better knowledge of the telemedicine system.

### Data analysis

Data from the questionnaire’s responses and interviews were translated into English, then coded in Microsoft Excel. Next, a thematic analysis was undertaken to identify the important themes from the participants’ response in the three open-ended questions of the questionnaire and clinicians’ comments from the interview. The participants’ comments were initially classified in categories of the questionnaire examined: positive aspects; negative aspects, and suggestions for the future improvement of telemedicine.

## Results

One hundred and thirty questionnaires were distributed to the 39 primary health centres based on the number of clinicians using the system in each site, and 100 were received back. Some clinicians from the health centres were off duty at that time or were not interested in completing the questionnaire. Fifteen of the 100 participants were chosen for in depth interviews, which lasted on average of 31.3 min.

Each clinic site initially had 2 clinicians trained to use the system and they could train additional colleagues locally if required. The 100 completed questionnaires (76.9% response rate) represented a mean of 2.56 questionnaires per clinic, including some clinicians trained locally, suggesting a high level of interest in the survey.

### Clinicians’ perceptions in using telemedicine from the survey

The first part of the questionnaire consisted of seven questions. This part was to assess four aspects: (1) training (question number 1), (2) ease of use (questions number 2 and 3), (3) perceived usefulness (questions number 4 and 5), (4) satisfaction and continuous intention to use (questions number 6 and 7). (See Table 1).

**Table 1:** Results of part one of the questionnaire

Domain Examined	Frequencies (%) ***n*** = 100		
	Yes	No	No response
1. Have you ever received any training in telemedicine?	64%	32%	4%
2. Is the system easy to use and navigate?	71%	23%	6%
3. Is the information presented clearly?	70%	22%	8%
4. Is telemedicine beneficial for your patient?	89%	8%	3%
5. Does telemedicine provide desirable results in patient diagnoses?	85%	12%	3%
6. Overall, are you satisfied with the system?	78%	15%	7%
7. Are you interested to continue to use the telemedicine system?	88%	9%	3%

The Table 1 summarised the percentage of each question from four aspects evaluated in this first part of the questionnaire. 78% of the clinicians expressed satisfaction with the system and 88% expressed interest in continuing to use it. Based on this, the first part of the questionnaire suggested a positive perception of the telemedicine system.

The second part of the questionnaire consisted of two questions to examine clinicians’ satisfaction level in the field of equipment and procedure, and the technical quality of the service. (See Table 2).

**Table 2:** Results of part two of the questionnaire

Domain Examined	Frequencies (%) ***n*** = 100			
	Satisfied	Neutral	Dissatisfied	No response
1. How do you rate the comfort of using telemedicine equipment and procedures?	77%	18%	0	5%
2. How do you rate the technical quality of the service?	54%	42%	0	4%

Table 2 summarised the second part of the questionnaire that assessed clinicians’ satisfaction in using the equipment and technical quality of the service. The clinicians’ satisfaction levels in using the telemedicine equipment were high at 77%. For technical quality 54% were satisfied, but the remaining 42% chose neutral.

### Clinicians’ satisfaction based on qualitative data

This study identified five factors that the clinicians liked most. (See Table 3)

- Making faster diagnosis
  *“Every time I found a confusing case, I referred a patient to a hospital soon. But, now I can contact the specialist through telemedicine and receive the second opinion soonest. I think this is the best benefit I find from the system.”* (General Practitioner, Primary Care).
- Reduce referrals
  *“The number of referrals reduced significantly since we use telemedicine. Now, I can refer no cases to a hospital since I can consult with the specialist immediately through this system.”* (Head of the primary care, Primary Care).
- Increase patients’ trust
  *“It is quite funny, but although the patients do not know the name of the system, but seeing us using high technology equipment, it makes them having more trust in us.”* (General Practitioner, Primary Care)
- Improve skill and coordination
  *“I feel happy that I can learn more deeply on how to diagnose a more complex case with direct supervision from the specialist doctor. Besides, coordination with the specialist grows better.”* (General Practitioner, Primary Care).
- Easy to use
  *“Although it is quite new for me, I can operate it without difficulty since the feature is quite simple.”* (Nurse, Primary Care).

**Table 3:** Factors identified by the participants in the three open-ended questions in the questionnaire

Question	Factors identified	Total number of responses
**Like**	• Diagnosis and treatment faster	69%
	• Reduce referrals	3%
	• Increase patient’s trust	9%
	• Improve skill and coordination	4%
	• Easy to use	13%
**Concern**	• Technical quality is poor	47%
	• Increase workload and time consuming	18%
	• Limited funding	17%
**Suggestion**	• Improve infrastructure	40%
	• Improve service quality	34%
	• Periodical training	16%
	• Increase funding	10%

### Clinicians’ concerns based on qualitative data

This part identified four themes. (See Table 3)

- Technical quality is poor
  It involved a disruption of the internet connection, system errors, inappropriate and poor-quality equipment as well as the unavailability of electricity in the primary care clinics in the islands. These were considered as the main obstacles by most of the clinicians.
  *“Until today, I cannot provide telemedicine service regularly since the system in the health center I work cannot connect to the internet due to frequent connection disruptions.”* (General practitioner, Primary Care).
- Increase workload and time-consuming
  The increase of workload was the second challenge frequently mentioned by the clinicians, which affected their willingness to use telemedicine since it increased their working time. The interviewees mentioned that to set-up telemedicine equipment was complicated and time consuming, while there were many patients waiting to have the service. The clinicians even had to compile a report which according to them was confusing because there was no clear reporting standard.
  Sometimes, when technical problems occurred, the clinicians had to solve this problem themselves because there was no technical expert ready in the primary care clinics. *“Many time I try to use the telemedicine but technical problems frequently arise. I am a professional, but I have to handle technical things which are outside of my knowledge and usually, it takes rather long time while there are many patients waiting for me. It is so frustrating. I hope that I can only focus to serve patients as a physician.”* (General Practitioner, Primary Care).
- Limited funding
  It involved the operational cost related to telemedicine and remuneration for clinicians providing telemedicine services. Lack of special funding for the operation was considered to be one of the obstacles in providing the service, especially in the islands where electricity was only turned on during evenings, while activities during daytime had to use a gasoline generator. As a result, the expenses of the operation were very high in this area. Funding for the daily telemedicine service is usually taken from the operational budget of the primary care clinic, so the rest of the funding should be managed well so as to cover other service costs. This caused very tight budgeting. *“The operational cost for telemedicine is quite expensive, so I have to arrange the budget properly to make all service run well.”* (Nurse, Primary Care).
  In addition, some interviewees said that there was no remuneration for the primary care clinicians providing this service.

### Clinicians’ suggestions for improvement

This part identified five themes. (See Table 3)

- Improve infrastructure
  Some suggestions focussed on the infrastructure improvement for telemedicine, such as having an appropriate internet connection by establishing a cooperation with some communication providers, and also securing the availability of electricity in the island’s area.
  *“The first priority is to have a stable internet connection. It is very important. It is a must.”* (General Practitioner, Primary Care). The importance of having a private satellite dish for health services was also proposed by 5 interviewees. *“We need our own satellite to have a powerful system”* (General Practitioner, Primary Care).
- Improve service quality
  Most of the participants in the questionnaire suggested enhancements to the service quality, that is the fast delivery of the result from the specialist doctor. They also proposed to have an open communication channel with the specialist doctor, improving the content of features and simplifying the procedure.
  *“I will receive the result from the specialist in 1 or 2 hours, but sometimes I will receive it at night. So, it means that the patients have to go back to the primary care the day after to know the result and receive medication”* (General Practitioner, Primary Care). They assumed that this happened due to the lack of specialist doctors involved in this project. Furthermore, some clinicians suggested that the coordination between the clinicians in primary care and the specialist should be improved, but other clinicians noted that there was no supporting feature to make it possible to facilitate direct communication such as video consultation between them, and there were limited items that they could put in the patient’s history column. Therefore, comprehensive direct coordination was difficult to achieve. Another suggestion was the importance of simplifying the procedure and reporting system. Relating to the technical work, they emphasized that as health professionals, they were not obliged to do the technical work. They proposed to appoint at least one technician in every primary care clinic.
  Another suggestion was to expand the type of telemedicine services and establish a wider cooperation with many specialist doctors as well as the schools of medicine. Involving them in this service might overcome the long waiting time in receiving the diagnosis results.
  *“The tele-USG is not just to examine the pregnant woman, but it can be used to examine other parts of the body, so if we can use it for many cases, why not?!”* (General Practitioner, Primary Care).
- Periodic training
  Periodic operational training was recommended by 16 participants in the questionnaire in order to improve skills, whereas 4 general practitioners in the interview proposed to have an advanced training for reading the more complex examination result of the ECG and USG.
  ***“****In order to refresh our skill and knowledge, especially for the clinicians who did not use the system regularly.”* (General Practitioner, Primary Care).
- Increase funding
  The clinicians thought that remuneration would become a good motivation for the health workers to give their best service since they also received attention for their welfare. *“Remuneration for clinicians would be very good. It means that the Government pays attention to us and it will motivate us to work better”* (General Practitioner, Primary Care).
  Besides, operational cost for the daily operation was also important.
  *“It is a free service for telemedicine patient, but operational cost such as electricity are not free. The importance to provide daily operation cost is undeniable.”* (Nurse, Primary Care).

## Discussion

To the best of the author’s knowledge, this study is the first telemedicine study capturing the clinicians’ point of view and experiences in Makassar, and in Indonesia generally. Overall, the result indicated that the clinicians were satisfied with the telemedicine system, and almost all of them were interested in continuing using this system since they considered that telemedicine was beneficial for their practice.

The advantages reported in this study include getting faster diagnosis, reducing unnecessary referrals, improving health workers skill, and increasing patient’s trust. These are in line with published studies (see Additional file 3). A randomised control trial by Whited et al. on teledermatology reported that 92% of the referring clinicians and 75% of the dermatologist consultants were satisfied with the system, and 95% of the referring clinicians stated that teledermatology allowed more timely referrals for their patients [11].

Many prior studies had shown the excitement and interest of clinicians to use telemedicine (see Additional file 3), but in many locations and disease areas this expectation had not been achieved yet. Many challenges are summarised by Kruse et al. in a systematic review about the barriers in adopting telemedicine worldwide [12]. Technical barriers, resistance to change, licensing issues, perception of impersonal care, and information overload were the most common barriers identified by staff [12]. In this study, we found that in the primary care clinics on the islands, grid electricity was only turned on in the evening time, so all activities during daytime had to use a generator. In addition, there are limited Base Transceiver Stations located near to these islands, which limited communication and sometimes causes outages. These factors were clearly shown to discourage use of telemedicine, particularly in remote sites that might have higher benefits from the service.

Based on the above, clinicians in this study suggested infrastructure improvement in telemedicine, such as by establishing a cooperation with some communication providers to have a stable internet network and securing the availability of electricity in the island’s area. As an alternative, using the solar energy as a low-cost way of supplying power in the day time for primary care sites could be considered.

Clinicians also made suggestions to establish coordination with many specialist doctors and the schools of medicine, to create a reporting standard, and add a supporting feature in the system to enable the clinicians to engage in direct interaction with the specialist through video consultations to improve the coordination.

Mixed methodology was used in this study to combines elements of quantitative and qualitative research to increase the breadth and depth of understanding [13]. Tashakkori et al. defined this method as a research in which the investigator collected and analysed data, integrated the findings, and found conclusion using both qualitative and quantitative approaches [14]. Quantitative data can support the qualitative research component by identifying outlying cases, while qualitative data can help the quantitative components to develop the conceptual model or instrument. During data analysis for this study, qualitative data was used to assist with interpreting, clarifying, describing, and validating the quantitative results [13].

Several studies have investigated clinicians’ perceptions on the use of telemedicine. However, the majority of this research was conducted in United States, Europe and other developed countries, with a limited number of studies of this type conducted in developing countries (see Additional file 3).

Research conducted by Pasco et al. in the Philippines, a country with geographic characteristics identical to Indonesia, shows the need for better infrastructure [15]. This result is similar to our finding, although the number of respondents is smaller. At present, there is no other data on clinicians’ perceptions of telemedicine in Indonesia. Therefore this study provides a basis for understanding the potential for wider use in this large country, and in other developing countries in areas with characteristics similar to Indonesia such as a large number of islands.

Baruffaldi et al. in a research in Bologna and Johansson et al. in Sweden found high satisfaction with video consultation [16, 17]. Most physicians preferred to use asynchronous telemedicine since it was easily integrated in the practice, however clinicians had less confidence in their diagnosis when using this type of telemedicine [16, 17]. This was because of the lack of real-time feedback from an expert and the poor quality of information transmitted in many cases that could affect the decision made in diagnosis and treatment. Even though real time video consultation required more complex equipment, it was clearly preferred for more for specialized cases [16].

Periodic training was considered to be very important in order to refresh clinicians skills and maintain a proper service, as the lack of clinicians’ knowledge of telemedicine would be the greatest barrier of the implementation and adoption [18, 19]. In a research study on 130 nurses that had been given hands-on training in telemedicine on weekly basis, Brebner et al. from the North West Regional Telehealth Resource Centre (NRTRC) in the United States proved that the level of user competence could reach 100%, as well as a high level of satisfaction [20]. Thus, by demonstrating telemedicine capabilities and advantages, it could increase their confidence and encourage them to use this technology [6]. Relating to the remuneration, the clinicians proposed that the local government should offer remuneration to the clinicians who provided this service and provide special funding for the operation of telemedicine. Unfortunately, they did not give recommendations on how the government should provide this fund.

There is extensive literature regarding telecardiology and teleultrasound diagnosis effectiveness. Vodicka et al. in a systematic review regarding telecardiology in Slovenia concluded that telecardiology significantly reduces the number of unnecessary referrals to a cardiologist or hospitalization, and shortens the time needed to treat patients with life-threatening conditions [21]. Britton et al. in a systematic review showed that teleultrasound provided satisfactory quality and value for clinical diagnosis and management [22].

Indonesia is a classic example of a country where low numbers of specialist, uneven distribution of clinician and geographic barriers frequently prevent access to specialist care. This study shows successful uptake and use of a locally developed telehealth system in both a main island and a separate island. We believe this experience should be generalizable broadly in Indonesia including (with modifications) for COVID-19 screening. As planned by the local government for the future, this telehealth platform can be expanded to other types of ultrasound and could be used for other image-based diagnosis including dermatology and ophthalmology and capture of plain x-rays using a digital camera or scanner.

This study emphasized the role of perceptions of usefulness concerning telemedicine acceptance in primary care. This result is consistent with other previous studies stating that the perception of usefulness would influence individual acceptance and use of a technology (see Additional file 3) [23–26]. The significant result of this study showed that if health workers realize the usefulness of telemedicine, they will accept this system more easily and have continuous intention to use it for their daily practice. It will likely increase clinician retention in using telemedicine [5, 26]. In addition, this study aims to inform and encourage the decision makers to make continuous efforts to improve the perception of usefulness of the system for health workers, since this would likely encourage the implementation, deployment, and sustainability of the system.

## Limitation

This study has some limitations. First, the response rate in this study was 76.9%, however this was in excess of the number expected if the 2 originally trained staff per site responded. Second, there is a lack of direct measure of number of referrals actually occurring before and after system was implemented. Third, some of the responders expressed discomfort and reluctant in giving their opinions in this survey. They may have considered saying positive things to support this project (desirability bias). The research sought to examine clinicians’ perception of the telemedicine implementation and enablers and barriers to use, and did not look into specific issues relating to any specialism in telemedicine or measures of diagnostic or referral accuracy.

## Conclusion

The findings from this study indicated that the clinicians were satisfied with the system, with the main benefits seen to be rendering the diagnosis faster. However, technical problems such as the internet connectivity and availability of electrical power were indicated as the main barriers which led to the suggestion to improve the infrastructure. Future work needs to examine the diagnostic accuracy achieved by the system and the impact on patient referrals, travel, and outcomes.

## Supplementary information

**Additional file 1.** Total tele-ECG cases in 2017.

**Additional file 2.** Number of telemedicine cases per month in primary care.

**Additional file 3.** Comparison of telemedicine studies.

## Data Availability

The datasets used and/or analysed during the current study are available from the corresponding author on reasonable request.

## Abbreviations

WHO: World Health Organization
Tele-ECG: Tele-electrocardiography
Tele-USG: Tele-ultrasonography
