# Supplementary material for: Clinicians perceptions of a telemedicine system: a mixed method study of Makassar City, Indonesia
Source: BMC Med Inform Decis Mak. 2020 Sep 17;20:233. doi: 10.1186/s12911-020-01234-7 (PMC7495970; doi:10.1186/s12911-020-01234-7)
Supplement: Supplementary file 1 — Additional file 1. Total tele-ECG cases in 2017. [file 12911_2020_1234_MOESM1_ESM.docx]

**Additional File 1**

Total tele-ECG cases in 2017

| **No.** | **Month** | **Total Tele-ECG Cases** |
| --- | --- | --- |
|  | January | 315 |
|  | February | 378 |
|  | March | 367 |
|  | April | 470 |
|  | May | 473 |
|  | June | 201 |
|  | July | 280 |
|  | August | 294 |
|  | September | 282 |
|  | October | 405 |
|  | November | 283 |
|  | December | 226 |
|  | **TOTAL** | **3974** |
