# Supplementary material for: Clinicians perceptions of a telemedicine system: a mixed method study of Makassar City, Indonesia
Source: BMC Med Inform Decis Mak. 2020 Sep 17;20:233. doi: 10.1186/s12911-020-01234-7 (PMC7495970; doi:10.1186/s12911-020-01234-7)
Supplement: Supplementary file 2 — Additional file 2. Number of telemedicine cases per month in primary care. [file 12911_2020_1234_MOESM2_ESM.docx]

**Additional File 2**

Number of telemedicine cases per month in primary care

| **Primary Care** | **Approximate telemedicine cases per month** | **Number of populations** |
| --- | --- | --- |
| Primary care 1 | 7-10 | 19,144 |
| Primary care 2 | 8-12 | 14,743 |
| Primary care 3 | 10-15 | 13,246 |
| Primary care 4 | 4-6 | -- |
| Primary care 5 | Around 5 | 24,807 |
| Primary care 6 | Around 5 | 39,812 |
| Primary care 7 | Around 5 | 70,955 |
| Primary care 8 | Around 3 | 32,403 |
| Primary care 9 | 5-10 | 22,311 |
| Primary care 10 | 3-4 | 14,721 |
| Primary care 11 | 4-6 | 14,918 |
| Primary care 12 | -- | -- |
| Primary care 13 | Around 10 | 37,156 |
| Primary care 14 | 7-10 | 22,933 |
| Primary care 15 | 4-8 | 22,389 |
| Primary care 16 | -- | -- |
| Primary care 17 | -- | -- |
| Primary care 18 | Around 6 | 19,322 |
| Primary care 19 | 5-7 | 16,819 |
| Primary care 20 | Around 5 | 20,267 |
| Primary care 21 | 5-7 | 80,665 |
| Primary care 22 | Around 5 | -- |
| Primary care 23 | Around 10 | 40,980 |
| Primary care 24 | 3-4 | 11,683 |
| Primary care 25 | -- | -- |
| Primary care 26 | 5-10 | -- |
| Primary care 27 | -- | -- |
| Primary care 28 | -- | -- |
| Primary care 29 | 7-10 | 66,202 |
| Primary care 30 | 5-7 | -- |
| Primary care 31 | Around 5 | 43,329 |
| Primary care 32 | Around 10 | 48,777 |
| Primary care 33 | Around 7 | 23,409 |
| Primary care 34 | 5-10 | 25,411 |
| Primary care 35 | 5-10 | 18,923 |
| Primary care 36 | -- | -- |
| Primary care 37 | Around 5 | -- |
| Primary care 38 | 5-7 | 67,014 |
| Primary care 39 | 7-10 | -- |
| Primary care 40 | Around 5 | 102,326 |
| Primary care 41 | 5-10 | -- |
| Primary care 42 | 5-10 | 34,398 |
| Primary care 43 | Around 10 | 17,544 |
| Primary care 44 | 7-10 | 35,616 |
| Primary care 45 | 7-10 | -- |
| Primary care 46 | 7-10 | 16,617 |

Key

- Less than 4 cases per month = 3 sites (red colour)
- 5-10 cases per month = 34 sites (black colour)
- 10+ cases per month = 2 sites (green colour)
