# Supplementary material for: Clinicians perceptions of a telemedicine system: a mixed method study of Makassar City, Indonesia
Source: BMC Med Inform Decis Mak. 2020 Sep 17;20:233. doi: 10.1186/s12911-020-01234-7 (PMC7495970; doi:10.1186/s12911-020-01234-7)
Supplement: Supplementary file 3 — Additional file 3. Comparison of telemedicine studies. [file 12911_2020_1234_MOESM3_ESM.docx]

**Additional File 3**

Comparison of telemedicine studies

| **Author** | **Country** | **Telemedicine** | **Methods** | **Results** |
| --- | --- | --- | --- | --- |
| Whited et al (2004) | United States | Teledermatology | Survey as part of randomized clinical trial | - A majority of referring clinicians (92%) and dermatologist consultants (75%) satisfied with the teledermatology process. - 95% percent of referring clinicians reported that teledermatology provides quicker referrals. |
| Kruse et al (2018) | Worldwide | Not specified | Systematic review | - Identified issues related to technically challenged staff (11%), resistance to change (8%), cost (8%), reimbursement (5%), age of patient (5%), and level of education of patient (5%). - The top barriers are technology- specific and could be overcome through training, change-management techniques, and alternating delivery by telemedicine and personal patient-to-provider interaction. |
| Pasco (2016) | Philippines | Teleconsultation | Questionnaire and focus group discussion | - Social influence was the most important factor that influenced clinician intention to use telemedicine. - Barrier: Internet connectivity was a problem in many areas, poor telecommunication infrastructure, the need to have available specialists to answer referrals, having technical staff to respond technical concerns. - Recommendation: funding for procuring equipment and incentive for physicians to increase interest to use and promote telemedicine services. |
| Baruffaldi et al (2002) | Italy | Teleconsultation for orthopaedics | Questionnaire | - Both the referring clinicians and orthopaedic specialists were satisfied with the system. - The majority choose asynchronous telemedicine because it could easily integrate into clinical practice. However, the clinicians' confidence in diagnosis was less for the asynchronous. - Videoconferencing was preferred in more demanding cases. |
| Johansson et al (2017) | Sweden | Video consultation for dermatology and cardiology | Mixed methods: questionnaire and interview | - Overall satisfied with the system, because telemedicine provides quicker contact with the specialist, learning opportunity and increase knowledge for the referring clinicians, the referrals have decreased and save time. - Video consultation was a cost- effective solution for rural areas. - The barrier: specialists cannot touch patient’s skin lesion and inconvenient if the patient had to wear headphones because of impaired hearing. - Recommendation: the technology should be simplified, and make sure that the workplace is ready for the new working method by training and educating the staff. |
| El-Mahalli et al (2012) | Saudi Arabia | Not specified | Cross-sectional descriptive study | - Benefit: improving the quality of care, enhancing access to healthcare, and providing patient care and management. - The barrier: as perceived by health providers was the lack of knowledge about telemedicine. - Recommendation: dissemination of information about telemedicine and proper training of health professionals. |
| Brebner et al (2003) | United Kingdom | Videoconferencing | Questionnaire | - High levels of satisfaction were obtained but the level of user competence reached 100% only when training was supported by a training manual and at least weekly practice. For those who had the opportunity to practise only monthly, their competence level dropped to 60%. For those who had the opportunity to practise less often than monthly, their competence level dropped to 43%. |
| Vodicka et al (2020) | Slovenia | Telecardiology | Systematic review | - Benefit: use of telemedicine can shorten the time from diagnosis to the necessary treatment, reduce mortality in patients with acute myocardial infarction, shorten the time to diagnose atrial fibrillations and help determine the diagnosis for patients complaining about heart rhythm disorders which were not detected on the standard ECG recording, also help identify cardiac causes for syncope or collapse. - The use of telecardiology significantly reduces the number of unnecessary referrals to a cardiologist or hospitalization, and shortens the time needed to treat patients with life-threatening conditions. - The use of telecardiology increases the quality and safety of work in managing patients with cardiovascular disease in family physician practice. - Usage of telecardiology devices can also save money and bridge the gap between the primary and secondary healthcare levels. |
| Britton et al (2019) | Worldwide | Tele-ultrasound | Systematic review | - Overall, tele-ultrasound in resource-limited settings are of satisfactory and value for clinical diagnosis and management. |
| Davis (1989) | Canada and United States | - | Field and lab study | - Perceived usefulness was significantly correlated with both self-reported current usage and self-predicted future usage. - Perceived ease of use was also correlated with current usage and future usage. |
| Venkantesh (2000) | United States | - | Longitudinal field studies | - Individual’s general beliefs regarding computers were the strongest determinants of system-specific perceived ease of use. - The need for an increased focus on individual difference variables in order to enhance user acceptance and usage. - The need to develop and implement general training programs on computer skills as they will have a strong influence on the acceptance and sustained usage of new systems. |
| Chintakovid (2007) | Not specified | - | Experimental design | - This study revealed that intention to use a system is affected by extrinsic motivation (perceived usefulness) and intrinsic motivation (perceived ease of use). |
| Zailani et al (2014) | Malaysia | Not specified | Quantitative survey | - This study suggests that government policies, top management support, perception of usefulness and computer self- efficiency have a significant impact on telemedicine acceptance. - Important relationship between government policies as well as perceived usefulness on telemedicine acceptance. |
